# Frozen-in condition for ions and electrons: implication on magnetic flux transport by dipolarizing flux bundles

**DOI:** 10.1186/s40562-018-0104-0

**Published:** 2018-02-10

**Authors:** A. T. Y. Lui

**Affiliations:** JHU/APL, Laurel, MD 20723-6099 USA

## Abstract

The ability of dipolarizing flux bundles (DFBs) in transporting magnetic flux from the mid-tail reconnection site for near-Earth dipolarization is evaluated by two methods: the generalized Ohm’s law and the concept of flux preserving and line preserving. From the generalized Ohm’s law, the breakdown of the frozen-in condition (FIC) for ions is shown to be intimately related to that for electrons. When FIC is not satisfied for the ion fluid associated with energy conversion, it also implies the same for the electron fluid. When FIC holds, the plasma has the flux preserving property. It further guarantees that charged particles on a given magnetic field line will stay together on a magnetic field line at later times, i.e., line preserving. Conversely, when line preserving does not hold, flux preserving does not hold also. Previous detailed examination on the FIC for DFBs revealed that the majority of DFBs associated with energy conversion violate the FIC for the ion fluid. This implies that FIC does not hold for the electron fluid also. Furthermore, plasmas in substorm injections come from vastly different locations, violating the line preserving property and implying that FIC is broken for the magnetic flux tubes associated with substorm injection and dipolarization. These observations indicate that DFBs are not an effective agent to transport magnetic flux within the magnetosphere and further imply that mid-tail magnetic reconnection is rather ineffective in transporting magnetic flux for near-Earth dipolarization.

## Background

Magnetic field reconfiguration, commonly referred to as dipolarization, is frequently observed with plasma injection in the near-Earth region (*X*_GSM_ > − 15 *R*_E_) for typical magnetospheric substorms (e.g., Akasofu 1968; DeForest and McIlwain 1971; McPherron et al. 1973). One common idea of linking mid-tail magnetic reconnection at *X*_GSM_ ≈ − 20 *R*_E_ to near-Earth dipolarization is through the consideration of magnetic flux transport by plasma flows from magnetic reconnection. Magnetic structures associated with Earthward reconnection flows have been observed to exhibit transient large northward swings in the magnetic field called dipolarization fronts (DFs) (Nakamura et al. 2002; Runov et al. 2009, 2011; Schmid et al. 2011). Liu et al. (2013, 2014) studied the magnetic flux transport associated with DFs, referring them as dipolarizing flux bundles (DFBs) and proposing them as elementary elements for the substorm current wedge (SCW). In this viewpoint, DFBs are considered to transport magnetic flux from the mid-tail to the near-Earth region for SCW development. For this scenario to be realizable, DFBs must satisfy the criterion for visualizing magnetic flux within them to be carried by the plasma bulk flow, which is the criterion for the frozen-in condition (FIC).

In contrast to the above viewpoint, there are several proposed processes in which substorm dipolarization in the near-Earth region is produced close to the Earth (e.g., Lui et al. 1988; Lopez et al. 1988, 1989; Roux et al. 1991; Lui 1991, 2011, 2013; Cheng and Lui 1998; Henderson 1994, 2009; Liu 1997; Cheng 2004; Liu et al. 2012; Haerendel et al. 2012; Haerendel and Frey 2014; Akasofu 2013, 2017). For comparison between these two viewpoints, it is important to address how effective are DFBs in transporting magnetic flux from the mid-tail region to the near-Earth region.

## The frozen-in condition

The FIC was introduced by Alfvén (1942) to visualize the properties of the low-frequency electromagnetic Alfvén waves resulting from the combination of the hydrodynamic equations with Maxwell equations. It is expressed by the criterion $\varvec{E} + \varvec{V} \times \varvec{B} = 0$, where ***E*** is the electric field, ***V*** is the plasma bulk velocity, and ***B*** is the magnetic field. The validity of this condition allows one to visualize transport of magnetic flux through the plasma fluid motion. If this condition does not hold, then magnetic field line motion is not applicable, since it is ill-defined as it does not tie to the plasma fluid motion. As a result, magnetic flux transport cannot be visualized to be carried by the plasma fluid motion. Therefore, the ability of DFBs to transport magnetic flux from the mid-tail to the near-Earth region requires the validity of the FIC along their entire paths.

Whether or not the FIC is satisfied can be examined with the generalized Ohm’s law, which is essentially the electron momentum equation. Adopting the good approximation that the ion fluid velocity ***V***_i_ represents well the plasma bulk velocity, this law can be written in International system (SI) units as (e.g., Parks, 2004, p. 296):

1 $$\varvec{E} + \varvec{V}_{\text{i}} \times \varvec{B} = \frac{{\varvec{J} \times \varvec{B}}}{ne} + \varvec{D} ,$$

where ***J*** is the current density, *n* is the number density, *e* is the elementary electric charge, and ***D*** denotes the sum of terms associated with non-ideal magnetohydrodynamics (MHD) effects from inertial, electron viscosity, and anomalous resistivity. If the FIC is broken only by the first term on the RHS (the Hall term), then there is no energy conversion (dissipation or dynamo), because the triple product ***J***·***J*** × ***B*** is precisely zero. Without energy conversion, DFBs lack the ability to have dynamo effect to drive field-aligned currents and to be a part of an elementary SCW. Furthermore, they cannot energize particles, because no dissipation is involved in this situation. Therefore, for DFBs to possess these properties, the breakdown of FIC for DFBs must involve ***D*** ≠ 0.

By moving the Hall term on the RHS to the LHS and noting that ***J***/*ne* = ***V***_i_ ***−*** ***V***_e_, Eq. (1) becomes

2 $$\varvec{E} + \varvec{V}_{\text{e}} \times \varvec{B} = \varvec{D} ,$$

where ***V***_e_ is the electron bulk velocity. Therefore, for situation when DFBs exhibit dynamo effects and/or particle energization associated with breakdown of the FIC for the ion fluid, the FIC does not hold for the electron fluid also because ***D*** ≠ 0 in this situation. In other words, when energy conversion exists and the magnetic flux cannot be viewed as carried by the ion flow, then magnetic flux cannot be viewed as carried by the electron flow also. Furthermore, the dot product with ***J*** for Eqs. (1) and (2) gives the same value, that is

3 $$\varvec{J} \cdot \left( {\varvec{E} + \varvec{V}_{\varvec{i}} \times \varvec{B}} \right) = \varvec{J} \cdot \varvec{D} = \varvec{J} \cdot \left( {\varvec{E} + \varvec{V}_{\varvec{e}} \times \varvec{B}} \right) .$$

In other words, if there is dissipation or dynamo action in the frame of the ion fluid motion, there is a similar effect in the frame of the electron fluid motion. This point has been made by Lui et al. (2007) and Yao et al. (2017).

There is also another way to judge the validity of the FIC without involving direct measurements of the electric field or the fluid velocity or energy conversion consideration. When the FIC holds, it is called flux preserving and any magnetic flux tube moving with the plasma fluid velocity will enclose the same amount of magnetic flux with progress in time. Violating this obviously means that magnetic flux enclosed within the flux tube changes with time and thus cannot be viewed as travelling with the fluid velocity intact. Mathematically, it is expressed as

4 $$\nabla \times (\varvec{E} + \varvec{V} \times \varvec{B}) = 0,$$

where ***V*** is the fluid velocity. When all charged particles on a magnetic field line stay together on a magnetic field line at later times, it is called line preserving and is expressed as

5 $$\varvec{B} \times \left[ {\nabla \times (\varvec{E} + \varvec{V} \times \varvec{B})} \right] = 0.$$

Comparing Eqs. (4) and (5) indicates that flux preserving is a stricter condition than line preserving. Plasma that has flux preserving property guarantees to have line preserving property. Conversely, if line preserving does not hold, then flux preserving does not hold also (Newcomb 1958; Parks 2004, p. 188). This concept is based on the condition that a contour defining a magnetic flux tube will enclose the same amount of magnetic flux at later times when it moves with the fluid velocity. In this way, magnetic flux can be visualized to move with the fluid velocity.

Note that the concept of magnetic field line motion, leading to the idea that magnetic flux can be viewed as being transported by plasma fluid velocity, is based on a single fluid model. Therefore, the concept and the result of this work may not be applied to phenomena requiring a two-fluid or a kinetic description.

## Revealing observations

In this section, the theories presented in “The frozen-in condition” section are used to evaluate whether or not magnetic flux within DFBs can be visualized as being transported by the fluid velocity.

Runov et al. (2011) conducted a multi-case study of 18 cases of DFB. Energy conversion (dissipation: ***J***·***E*** > 0) took place in these events with clear indications of electron heating and increase in the high-energy electron flux. Subsequently, Lui (2015) examined these events in detail for the validity of the FIC for the ion fluid. It is found that the FIC for the ion fluid does not hold for 17 of the 18 cases (i.e., 94%). The FIC breakdown is determined by having the ratio of |[***E***_y_ + (***V*** × ***B***)_y_]/(***V*** × ***B***)_y_| exceeding 0.5, i.e., the mismatch of the two quantities exceeds 50%. This is a major discrepancy between the two quantities and cannot be ignored. Note that in his work, the electric field ***E***_y_ outside the DFB interval is calibrated by matching the averaged ***E***_y_ component with the averaged value of − (***V*** × ***B***)_y_ during appropriate quiet time of 5 min prior to DFB arrival. The plots provided in that study show excellent agreement between the two quantities prior to the encounter of DFBs. Variabilities of the electric field in each time interval are indicated by error bars in the comparison plot. Therefore, the judgement for the validity of FIC during DFB intervals has been made carefully. Since the FIC breakdown in DFB for the ion fluid description was associated with energy conversion, this condition applies also to the electron fluid description. As a result, the majority of DFBs cannot be considered as a means to transport magnetic flux from one region to another by both the ion velocity and the electron velocity.

It is well known that the charged particles have guiding center drifts that depend on the particle species and energy. Dispersive substorm injections are frequently observed. This feature arises from charged particles on the same magnetic field line at the onset of dipolarization become separated subsequently due to their different drifts, demonstrating the lack of the line preserving property for the injected population. Also, backward particle tracing for substorm injections by Gabrielse et al. (2012) have shown that the particle population for injections at the near-Earth region originates from a vast area with different locations as far downtail as *X*_GSM_ ≈  − 20 *R*_E_. In other words, the injected plasma does not have line preserving property, which in turn implies that it does not have flux preserving property either. This means that the FIC does not hold for the magnetic flux tube that is associated with substorm injection and dipolarization. If there are kinetic features in the injected population or in DFBs (e.g., Zhou et al. 2010), then they provide further evidence that the phenomenon cannot be described by a single fluid model and the description of magnetic field line motion and magnetic flux transport by the fluid velocity cannot be applied.

**Fig. 1:**
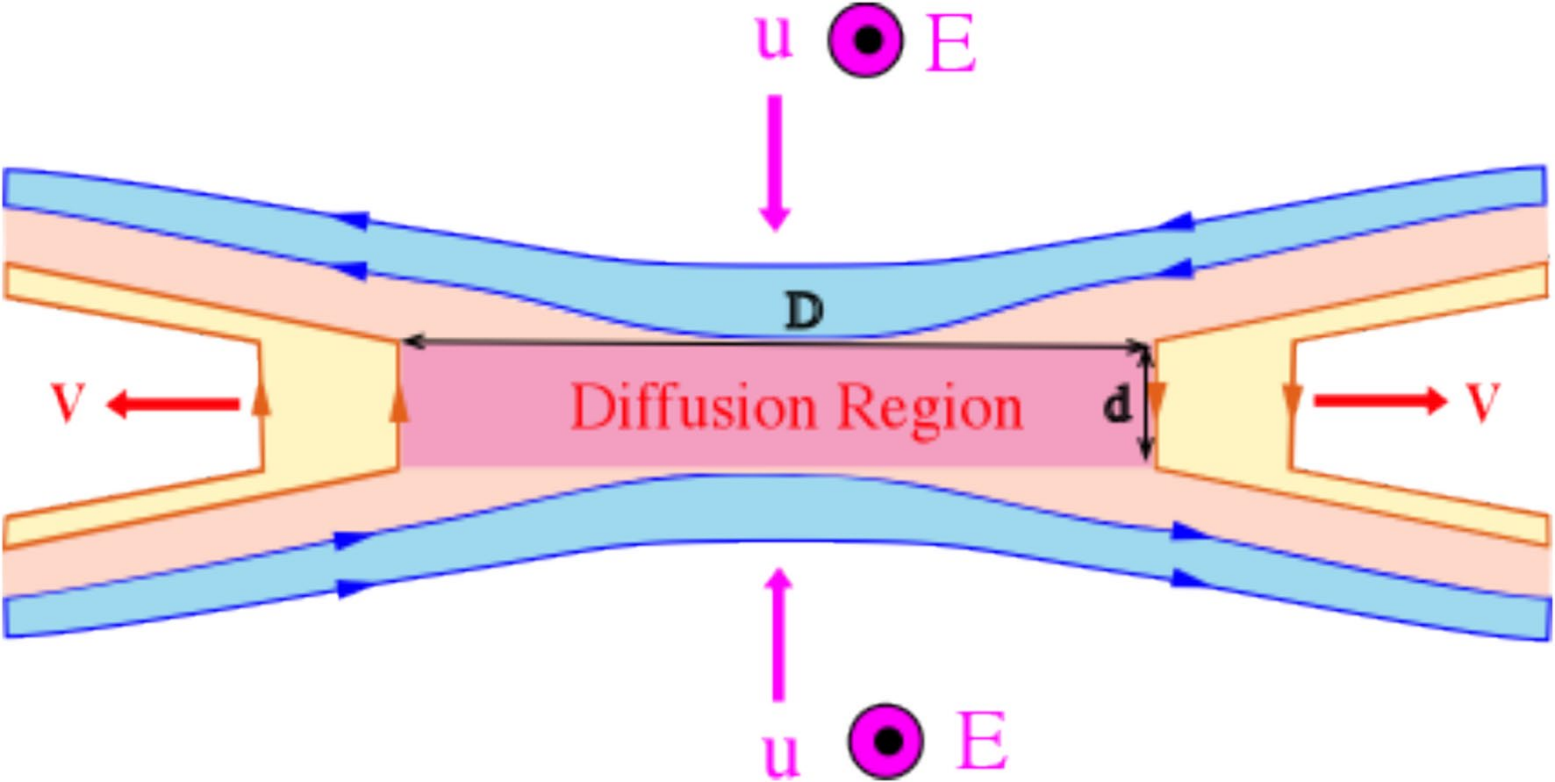
Case of a magnetic reconnection site is used to examine whether or not one can visualize a magnetic field line moving across the diffusion region can transport the same amount of magnetic flux in its exit. The mismatch between incoming and outgoing magnetic flux indicates no correspondence between incoming magnetic field line and outgoing magnetic field line

Further understanding on why magnetic field line (MFL) cannot be visualized to move across a region with FIC breakdown can be gained by considering the case of a magnetic reconnection site depicted in Fig. 1. Outside the diffusion region, the FIC holds, but in the region with MFLs passing through the diffusion region (the pink region), the FIC does not hold and MFL motion is not applicable. However, a question that can be raised is whether or not MFLs in the outflow region can be viewed as MFLs originating from the inflow region. This question can be answered by examining the situation quantitatively. Let *y* denotes the dimension of the diffusion region perpendicular to the reconnection plane. The amount of incoming magnetic flux *Φ*_1_ from the plasma inflow to the diffusion region per unit time from both sides is *Φ*_1_ = *2uB*_1_*yD*, where *u* is the inflow convection velocity, *B*_1_ is the magnetic field strength in the inflow region and *D* is the linear dimension of the diffusion region facing the plasma inflow. Since *u* = *E*/*B*_1_, where *E* is the convection electric field, then *Φ*_1_ = *2EyD*. Similarly, the amount of outgoing magnetic flux *Φ*_2_ in the plasma outflow per unit time from both sides is *Φ*_2_ = *2vB*_2_*yd* = *2Eyd*, where *v* is the outflow convection velocity, *B*_2_ is the magnetic field strength in the outflow region and *d* is the linear dimension of the diffusion region facing the plasma outflow. Since *D* > *d* for conversion of magnetic energy to particle energy in magnetic reconnection, then *Φ*_1_ > *Φ*_2_. Note that if *D* = *d*, there is no energy conversion. The magnetic flux reduction in the outflow region arises from energy conversion in the diffusion region. If we associate a finite magnetic flux *ϕ* for a MFL, then the number of incoming MFL is *Φ*_1_/*ϕ* and the corresponding number of outgoing MFL is *Φ*_2_/*ϕ*, which is less than the number of incoming MFL per unit time. Therefore, there is no continuity of magnetic field line across a diffusion region where the FIC is invalid, implying that there is no correspondence between MFL in the inflow region with that in the outflow region. Arbitrarily imposing equal number for incoming and outgoing MFLs will make them have different magnetic flux content. In other words, magnetic flux is not conserved in their transit through the diffusion region. Therefore, one cannot visualize MFL motion across a region where FIC is broken. Furthermore, particle transport by magnetic reconnection does not necessarily mean a complete magnetic flux transport associated with the particle motion.

## Summary and discussion

Two methods are used to evaluate the FIC for DFBs, namely, the generalized Ohm’s law and the concept of flux preserving and line preserving. From the generalized Ohm’s law, it is shown here that the breakdown of the FIC for the ion fluid is connected with the same condition for the electron fluid if the breakdown involves energy conversion. This interrelationship is not commonly recognized and led to the invalid argument that the FIC holds for the electron fluid even when it is violated for the ion fluid in spite of associated energy conversion. This perception comes from the thinking that electrons are magnetized because of their smaller gyroradius than that of ions and can be thought of as being tied to the magnetic field line. The present result dispels this misperception. The magnetized nature of electrons does not guarantee the validity of the FIC. Therefore, justifying magnetic flux transport of DFBs by claiming that electrons are still magnetized is not a valid argument.

Observations indicate that the plasma population associated with substorm injection and dipolarization does not have the line preserving property, implying also a lack of flux preserving property, i.e., the FIC is broken for the substorm injection population, reinforcing the result from the generalized Ohm’s law.

The present result should not be construed as an argument on the topic of MHD versus kinetic treatment in modeling magnetospheric dynamics correctly. This is not the key aspect of the work. It addresses only one particular phenomenon, i.e., DFB, and a particular issue, i.e., can magnetic flux be considered to be transported by DFB? If the FIC holds (as portrayed in MHD simulations), then obviously magnetic field lines can be visualized to move with the plasma fluid velocity and near-Earth dipolarization can be viewed as due to magnetic flux transport by DFB, such as shown in the MHD simulation of Wiltberger et al. (2015) and adopted by Kepko et al. (2015) for the SCW development. However, when the FIC is not satisfied, then magnetic field line motion cannot be identified with the plasma fluid velocity and in fact cannot be defined, which is the essence of what the FIC is about. This situation is sometimes referred to as magnetic slippage in which magnetic flux is visualized as slipping away from the plasma fluid motion. Therefore, the lack of FIC validity in most DFBs does not support the SCW development from mid-tail reconnection (e.g., Birn and Hesse 2014; Kepko et al. 2015). This is not surprising, since it has been shown that there is strong evidence for the near-Earth dipolarization to be a non-MHD phenomenon (Lui et al. 1999).

**Fig. 2:**
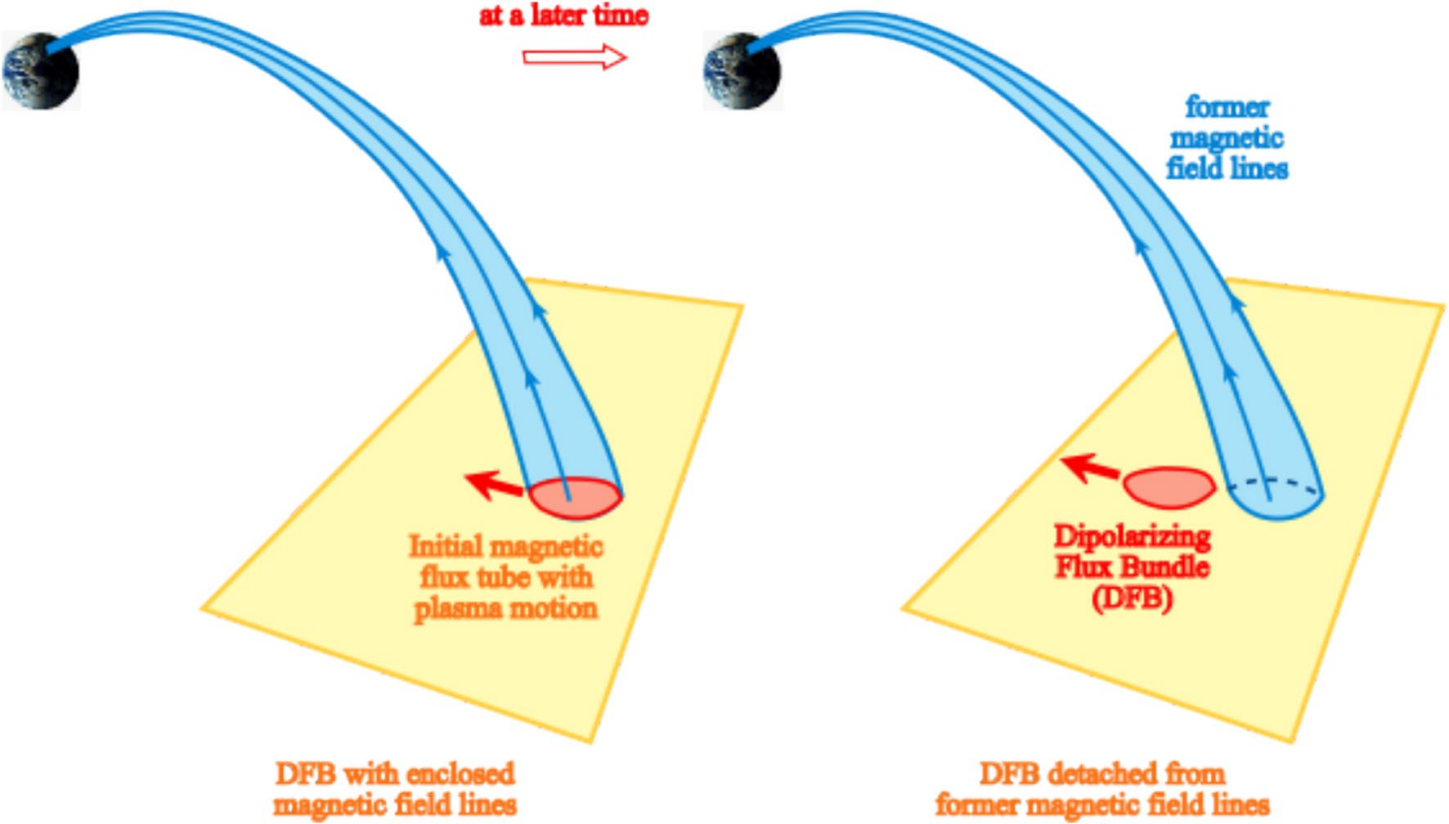
Schematic diagram to illustrate the lack of magnetic flux transport over a substantial distance by a dipolarizing flux bundle because of the severe departure from the frozen-in condition

Can there be a situation in which the ion fluid shows energy conversion and the FIC still holds for the electron fluid? From Eq. (1), because the triple product ***J***·***J*** × ***B*** is precisely zero, energy conversion can only occur when ***J***·***D*** ≠ 0, as discussed before. The situation of ***J***·***D*** ≠ 0 implies ***D*** ≠ 0; hence, $\varvec{E} + \varvec{V}_{\text{e}} \times \varvec{B}$ ≠ 0 from Eq. (2). In other words, the FIC does not hold for the electron fluid. This is a mathematically vigorous argument. Therefore, when the ion fluid exhibits energy conversion, the claim that the FIC still holds for the electron fluid violates the fundamental equation in fluid mechanics and cannot be valid. Furthermore, if the electron population originates from a source spread over a vast region as in DFBs (e.g., Gabrielse et al. 2012), then the electron plasma in DFBs does not have line preserving and flux preserving properties, indicating also that the FIC does not hold for the electron fluid and magnetic flux cannot be viewed as transported with the electron flow, consistent with the conclusion based on the generalized Ohm’s law. This situation is illustrated in Fig. 2 in which the magnetic flux associated with a DFB initially is detached from the DFB motion at a later time when there is a severe departure from the FIC.

Let us examine the viewpoint that DFBs without satisfying FIC can still transport part of the magnetic flux content with the plasma motion. The effectiveness of magnetic flux transport by DFBs can be evaluated with this viewpoint by estimating the amount of magnetic flux slippage for DFBs that do not obey the FIC. The validity of FIC for DFBs reported in the multi-case study by Runov et al. (2011) was evaluated by Lui (2015) using the comparison ratio *α* = |[***E***_y_ + (***V*** × ***B***)_y_]/(***V*** × ***B***)_y_|, with the result that *α* ranges from 0.6 to 11 when FIC is broken. This finding provides an opportunity to obtain a good estimate on how far DFBs that do not satisfy FIC can transport the magnetic flux content with the plasma motion. The total rate of change in magnetic flux content of a DFB is given by

6 $$\frac{{\varvec{d}{\varvec{\Phi}}}}{{\varvec{dt}}} = \mathop \int \limits_{\varvec{A}}^{{}} \frac{{\partial \varvec{B}_{\varvec{n}} }}{{\partial \varvec{t}}}\varvec{dA} + \mathop {\oint }\limits_{\varvec{l}}^{{}} \varvec{B} \cdot (\varvec{V} \times \varvec{dl}) .$$

The first term on the RHS is the effect of time variation of ***B*** over the cross section of the filament *A* and the second term is the change in magnetic flux content due to the motion of the boundary line *l* enclosing the DFB. Using the Faraday’s law on the first term and Stokes’ theorem on the second term, Eq. (6) becomes

7 $$\frac{{\varvec{d}{\varvec{\Phi}}}}{{\varvec{dt}}} = - \mathop \int \limits_{\varvec{A}}^{{}} \nabla \times \left( {\varvec{E} + \varvec{V} \times \varvec{B}} \right)_{\varvec{n}} \varvec{dA} .$$

The characteristic time *T*_c_ that the magnetic flux within the DFB can be transported with the plasma motion can be represented as

8 $$\frac{1}{{\varvec{T}_{\varvec{c}} }} = \left| {\frac{1}{{\varvec{\Phi}}}\frac{{\varvec{d}{\varvec{\Phi}}}}{{\varvec{dt}}}} \right| = \left| {\frac{1}{{\varvec{\Phi}}}\mathop \int \limits_{\varvec{A}}^{{}} \nabla \times \left( {\varvec{E} + \varvec{V} \times \varvec{B}} \right)_{\varvec{n}} \varvec{dA}} \right| = \left\{ {\frac{{\nabla \times \left( {\varvec{E} + \varvec{V} \times \varvec{B}} \right)}}{\varvec{B}}} \right\},$$

where {} denotes an estimate on the order of magnitude (Rossi and Olbert 1970, p. 286). Note that when $\varvec{E} + \varvec{V} \times \varvec{B} \to 0, T_{\text{c}} \to \infty ,$ as expected when the FIC holds. For DFBs in which the FIC is satisfied, they can indeed transport magnetic flux from the mid-tail region to the near-Earth region. For DFBs that have FIC broken, based on the work of Lui (2015), one may substitute ***E*** + ***V***×***B*** = *α*(***V*** × ***B***). In addition, since the gradient in the magnetic field appear abruptly at the leading edge of a DFB with a scale of *L*_g_, which is about the ion inertial length (Runov et al. 2009), then

9 $$\frac{1}{{\varvec{T}_{\text{c}} }} = \left\{ {\frac{{\nabla \times \left( {\varvec{E} + \varvec{V} \times \varvec{B}} \right)}}{\varvec{B}}} \right\} = \left\{ {\frac{{\varvec{\alpha V} \times \varvec{B}}}{{\varvec{L}_{\text{g}} \varvec{B}}}} \right\} = \left\{ {\frac{{\varvec{\alpha V}}}{{\varvec{L}_{\text{g}} }}} \right\}.$$

As *α* is of the order of 0.1–10, with ***V*** ~ 500 km/s, *L*_g_ ~ 500 km, *T*_c_ is then of the order of a fraction of a second or a few tens of seconds. This estimate matches with the abruptness in the changes of magnetic field with the encounter of a DFB. This indicates that the enhanced flux within a DFB can be transported by a distance of *VT*_c_ ~ a few tens to a few thousands km, which is small in terms of magnetospheric distance, especially in comparison with the distance between the mid-tail region and the near-Earth region. The efficiency of DFBs in transporting magnetic flux over long distances depends crucially on how often DFBs have FIC satisfied. Since DFBs having FIC broken with the ratio of |[***E***_y_ + (***V*** × ***B***)_y_]/(***V*** × ***B***)_y_| exceeding 0.5 is ~ 94%, their flux transport efficiency is extremely low. Although the sample of 18 cases is small, but the overwhelming preference for DFBs to break the FIC severely is undeniable. In other words, a very large number of DFBs is required to account for the magnetic flux in the near-Earth dipolarization for a moderate size substorm. This result was reached by Lui (2015) and Yao et al. (2015).

The result presented here has a significant impact to the scenario that mid-tail magnetic reconnection produces DFBs to transport magnetic flux to the near-Earth region for dipolarization there during substorm expansion. The distance separation between the two regions is ~ 10–20 *R*_E_. Without this means of magnetic flux transport, it seems necessary for dipolarization in the near-Earth region to be accomplished by some near-Earth processes, which may be MHD and/or kinetic processes. It is important to emphasize again that if the FIC holds for most of the DFBs, then mid-tail magnetic reconnection can indeed be viewed as providing the magnetic flux in near-Earth dipolarization through magnetic flux transport by DFBs. However, evaluating observations on this issue based on two different methods shows that the assumption of the FIC holding for most DFBs does not seem to bear out in nature.

## Abbreviations

DFs: dipolarization fronts
DFBs: dipolarizing flux bundles
FIC: frozen-in condition
LHS: left-hand side
MFL: magnetic field line
MHD: magnetohydrodynamics
NASA: National Aeronautics and Space Administration
NSF: National Science Foundation
RHS: right hand side
SCW: substorm current wedge
SI: International system
